# Identification of Pharmacokinetic Markers for Guanxin Danshen Drop Pills in Rats by Combination of Pharmacokinetics, Systems Pharmacology, and Pharmacodynamic Assays

**DOI:** 10.3389/fphar.2018.01493

**Published:** 2018-12-21

**Authors:** Hong Yao, Xiaomei Huang, Yunjiao Xie, Xuliang Huang, Yijun Ruan, Xinhua Lin, Liying Huang, Peiying Shi

**Affiliations:** ^1^Department of Pharmaceutical Analysis, School of Pharmacy, Fujian Medical University, Fuzhou, China; ^2^Department of Traditional Chinese Medicine Resource and Bee Products, Bee Science College, Fujian Agriculture and Forestry University, Fuzhou, China

**Keywords:** Guanxin Danshen drop pills, cardiovascular herbal medicines, pharmacokinetic marker, systems pharmacology, pharmacokinetics

## Abstract

This paper reported a feasibility study strategy of identifying pharmacokinetic (PK) markers for a cardiovascular herbal medicine, Guanxin Danshen drop pill (GDDP). First, quantification analysis revealed the constituent composition in the preparation by high-performance liquid chromatography (HPLC). Subsequently, physiochemical property calculation predicted the solubility and intestinal permeability of the constituents in the preparation. Furthermore, HPLC–MS analysis ascertained the absorbable ingredients and their PK properties in rat plasma. The main effective substances from the ingredients absorbed into blood and their cardiovascular effects were also predicted by systems pharmacology study, and were further confirmed by *in vivo* protective effects on isoprenaline-induced myocardial injury in mice. Finally, the ingredients with high content, representative structure feature, favorable PK properties, high relevant degree to myocardial ischemia (MI) issues, and validated therapeutic effects were considered as the PK markers for the preparation. Ginsenosides Rg_1_, Rb_1_, and tanshinone (TS) IIA were identified originally as PK markers for representing PK properties of GDDP. In addition, integrated PK studies were carried out according to previous reports, *viz*. drug concentration sum method and the AUC weighting method, to understand the *in vivo* process of GDDP comprehensively. The present study maybe provide a reference approach to identify PK markers for cardiovascular herbal medicines.

## Introduction

Traditional Chinese medicine (TCM) recipes have been used for several thousand years in Asia. Nowadays, although Western medicine has extensively supplanted TCM in modernized cities, TCM still plays an important role in Chinese health system (Stone, 2008; Liu et al., 2009). Considerable attention has been given to the usage of herbal medicine because many TCM remedies show effectiveness and less adverse effect in the treatment of some diseases, in which conventional Western medicine therapies fail or are proven insufficient to provide a palliative cure (Xue and Roy, 2003; Miller and Su, 2011). Currently, only few TCM products have been approved by Western public health systems (e.g., FDA) due to the deficiency of research data from modern science experiments for the herbal medicines (Hara, 2011). Furthermore, TCM is confronted with some unfavorable critics, e.g., assailing it as an outmoded folklore (Stone, 2008). To restore local and global belief in TCMs, evidence-based investigations for TCMs (including preclinical and clinical trials) should be performed. Among the modernization efforts, pharmacokinetic (PK) study is a crucial prerequisite to make TCM products evidence-based drugs because the dosage regimens, safety and potential herb–drug interactions require reasonable estimation at the systematic exposure to them.

However, unlike Western medicine pharmaceuticals, PK investigation on TCM products is really a challenge due to the complexity of constituents (numerous unknown and known chemicals coexisting in herbal medicines) and/or lack of suitable analytical methods. Up to date, researchers are still in the struggle to grope for comprehensive and reasonable strategies or methods to measure systemic exposure to complex TCMs. Under these circumstances, PK markers were put forward to deal with the PK issues of complex TCMs. Those active constituents possessing high contents and favorable PK properties (including a significant dose-dependent systemic exposure and an appropriate elimination half-life), could be considered as PK markers for herbal products (Lu et al., 2008; Hao et al., 2009; Shi et al., 2018). As a matter of fact, researchers desire to identify those characteristic ingredients which dominate the therapeutic effects of a TCM product *in vivo* due to their high bioactivity and favorable systemic exposure. Identification of components that should be considered representative index (PK markers) for PK evaluation of a TCM is a matter of debate. Idealistically, the selected representative PK markers for a multicomponent TCM should combine therapeutic effects and favorable PK properties to understand the relationship between administration and effect. To date, PK investigations on TCMs with consideration of the therapeutic effects of ingredients in TCMs have been conducted (Li et al., 2015; Yang et al., 2015; Zhou et al., 2015; Chang et al., 2016; Fan et al., 2016). Nevertheless, evaluation of the therapeutic effects of all unknown and known chemicals coexisting in a TCM is impractical. Therefore, more feasible strategies for identifying the representative PK markers for multiple-component TCMs should be proposed and verified; the strategies should focus on both the favorable PK properties and potent therapeutic effects for the identified markers.

**FIGURE 1:**
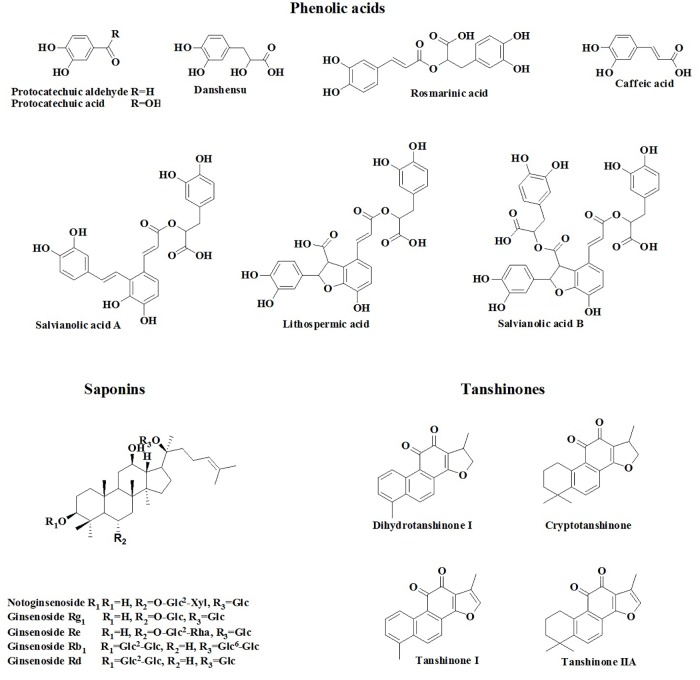
The structures of 17 ingredients in GDDP.

Cardiovascular disease (CVD) is the number one cause of death in the world. The World Heart Federation has reported that CVD accounts for 17.3 million deaths/year, and possibly 23.6 million deaths/year by 2030 (Smith et al., 2012; Hao et al., 2017). Because of the unmet needs for CVD control with Western medicine, clinicians is always considering the possible role of TCMs in the prevention and treatment of CVD, and a number of basic and clinical studies in this area have drawn increasing attention from the cardiovascular community (Hao et al., 2017; Lu et al., 2018). Among the considered TCMs, Guanxin Danshen drop pill (GDDP), a compound preparation prepared using three herbs, namely, *Salvia miltiorrhiza* (Danshen), *Panax notoginseng* (Sanqi), and *Dalbergia odorifera* (Jiangxiang), has been widely used to prevent and treat coronary artery heart disease in clinical practice in China (Tang et al., 2016). Pharmacological studies confirmed that GDDP could increase coronary blood flow (Fang et al., 2013), decrease myocardial oxygen consumption (Cai and Dai, 2013), ameliorate hemorheology property and heart function, and alleviate ischemia reperfusion injury (Tang et al., 2016). The cardiovascular therapeutic activities of GDDP could be mainly attributed to its anti-inflammatory, antioxidation, antiplatelet aggregation, and endothelial cell protection effects (Tang et al., 2016). Ginsenosides, salvianolic acids (SAs), and tanshinones (TSs) are regarded as the main active ingredients of GDDP (Tang et al., 2016). Our previous work developed the content determination method of 17 ingredients (Figure 1), including danshensu (DSS), protocatechuic acid (PCA), protocatechuic aldehyde (PCAL), caffeic acid (CA), rosmarinic acid (RA), lithospermic acid (LA), SA B (SAB), SA A (SAA), notoginsenoside R_1_ (R_1_), ginsenoside Rg_1_ (Rg_1_), ginsenoside Re (Re), ginsenoside Rb_1_ (Rb_1_), ginsenoside Rd (Rd), dihydrotanshinone I (DHTS I), cryptotanshinone (CTS), TS I (TS I), and TS IIA (TS IIA), in the preparations containing Danshen and Sanqi herbpair (Yao et al., 2017a). However, to date, there is no report on the PK profiles of the compound preparation. The PK properties of the active components in this preparation should be explored for clinical practice.

In this article, an approach to identify PK markers for the cardiovascular TCM preparation, GDDP, was proposed originally. The procedures and effectiveness of this method were verified and demonstrated in detail.

## Materials and Methods

### Chemicals and Reagents

DSS, PCA, PCAL, CA, RA, LA, SAB, SAA, R_1_, Rg_1_, Re, Rb_1_, Rd, DHTS I, CTS, TS I, TS IIA, and digoxin were purchased from Shanghai Ronghe Medicine Technology Development Co. Ltd. (Shanghai, China). The purities of all these references were greater than 98%. Guanxin Danshen dripping pills (GDDP, batch no. YR06524) were produced by Zhongfa Industrial & Commercial Group Yerui Pharmaceutical Co., Ltd., China according to a Chinese patent (CN200810145728.8) were purchased from a local drugstore (Fuzhou, China). Acetonitrile and methanol were of chromatographic grade (Merk KGaA, Darmstadt, Germany). Double distilled water was prepared in our lab. Glacial acetic acid was purchased from Sinopharm Chemical Reagent Co., Ltd. (Shanghai, China).

### Experimental Animals

The investigation conforms to the Guide for the Care and Use of Laboratory Animals published by the US National Institutes of Health (NIH Publication No. 85-23, revised 1996). The Animal Ethic Review Committee of Fujian Medical University (Fuzhou, China) approved all procedures. Male Sprague-Dawley rats (200 ± 20 g) and Kunming mice (about 8 weeks old and 20–30 g B.W.) were obtained from Laboratory Animal Center of Fujian Medical University, Fuzhou, China) were housed in rat cages (48 cm × 29 cm × 18 cm) and mouse cages (29 cm × 17.8 cm × 16 cm), respectively, in a unidirectional airflow room under controlled temperature (22 ± 2°C), relative humidity (40–70%), and a 12-h light/dark cycle. Filtered tap water was available *ad* *libitum*. The mice were given commercial mouse food *ad libitum.* The rats were given commercial rat food *ad libitum* except for the overnight period before dosing. All animals were acclimated to the facilities and environment for 7 days before the experiments.

### Assessment of Permeability and Solubility

Aqueous solubility (S) test of the 17 ingredients was performed by vortexing 10 mg of each ingredient in 10 mL water in a test tube at ambient temperature and 1 atm. After vortexing for 5 min, if the solution is clear, the tested object will be considered as soluble in water. *In silico* assessment of the physicochemical properties governing permeation and intestinal absorption was carried out for the 17 ingredients studied. Theory aqueous solubility (S) was calculated with ALOGPS 2.1 online software *via* the Virtual Computational Chemistry Laboratory service (Chemoinformatics Group, Neuherberg, Germany) (VCCLAB^1^). The octanol–water partition coefficient (cLog*P*), the total hydrogen bond count (donors and acceptors), and topological polar surface area (TPSA) were determined using the Molinspiration Property Calculator (Molinspiration Cheminformatics, Bratislava, Slovak Republic^2^). Lipinski’s rule of 5 was used to predict the permeability of the compound (Lipinski et al., 2001).

In addition, the bi-directional transport assay in Caco-2 cells for the mentioned ingredients in this paper has ever been carried out to evaluate their gastrointestinal absorption properties, and the reported bidirectional *P*_app_ [including *P*_app(basolateral→apical)_ and *P*_app(apical→basolateral)_] and efflux ratio [*P*_app(basolateral→apical)_/*P*_app(apical→basolateral)_] values were listed in Table 1.

**Table 1:** *In vitro* permeability in Caco-2 cell monolayers from literature and the results of *in silico* assessment of the 17 ingredients.

	*P*_app_ (×10^-6^ cm/s)	
Analytes	*P*_app(apical→basolateral)_	*P*_app(basolateral→apical)_	Solubility in water (mg/mL)	cLogP	TPSA	MW	nON	nOHNH
DSS	1.22^a^	0.903^a^	>10^e^	–0.25	97.98	198.17	5	4
PCA	4.52^b^	6.91^b^	>10^e^	0.88	57.53	154.12	3	2
PCAL	12.1^a^	21.8^a^	>10^e^	0.76	57.53	138.12	3	2
CA	4.35^b^	7.74^b^	>10^e^	0.94	77.75	180.16	4	3
RA	0.523^a^	0.550^a^	>10^e^	1.63	144.52	360.32	8	5
LA	0.0816^a^	0.0413^a^	>10^e^	1.57	211.28	538.46	12	7
SAB	0.0200^a^	0.0377^a^	>10^e^	1.61	278.04	718.62	16	9
SAA	0.0595^a^	0.0554^a^	>10^e^	3.01	184.97	494.45	10	7
R_1_	> 0.1^c^	> 0.1^c^	>10^e^	1.49	298.14	933.14	18	12
Rg_1_	> 0.1^c^	> 0.1^c^	>10^e^	2.77	239.22	801.02	14	10
Re	> 0.1^c^	> 0.1^c^	>10^e^	2.07	298.14	947.17	18	12
Rb_1_	> 0.1^c^	> 0.1^c^	>10^e^	0.28	377.29	1109.31	23	15
Rd	> 0.1^c^	> 0.1^c^	>10^e^	1.98	298.14	947.17	18	12
DHTS I	1.49^d^	8.28^d^	0.0077	3.51	43.38	278.31	3	0
CTS	1.59^d^	8.36^d^	0.0098	3.83	43.38	296.37	3	0
TS I	0.95^d^	11.27^d^	0.0115	3.83	47.28	276.29	3	0
TS IIA	0.98^d^	11.81^d^	0.0104	4.16	47.28	294.35	3	0


*^a-d^Mean that the data were collected from the previous reports by Lu et al. (2008), Bowles et al. (2017), Liu et al. (2009), and Hu et al. (2014), respectively. ^e^The values were experiment data in water at ambient temperature and 1 atm.*

### Administration and Sample Pretreatment for Multiple Component PK Study

Six rats were classified randomly into two groups, received a single p.o. administration of GDDP at 0.4 and 0.8 g/kg doses (the dosing solutions were prepared unifiedly into 1 mL with physiologic saline), respectively. Approximately 300 μL of blood was collected from the cut-tail before dosing and at the following time points 10, 15, 30, 45, 60 min, and 2, 4, 6, 8, 12, and 24 h after dosing. Plasma was isolated from the blood samples by centrifugation and then stored at -20°C until analysis.

For LC–MS analysis, samples stored were allowed to thaw at ambient temperature, followed by shaking for 30 s *via* a vortex apparatus. Plasma (100 μL) was removed and transferred to an appropriately labeled polypropylene tube (1.5 mL) containing 10 μL internal standard solution (5 μg/mL digoxin) and methanol (300 μL), followed by shaking for 2 min using a vortex apparatus. The samples were centrifuged (15,000 rpm for 10 min at 4°C) and 2 μL clear supernatant was injected into HPLC–MS system for bioanalysis by a validated HPLC–MS method.

### LC/MS Bioassays

Selection ion monitoring (SIM) and multiple reaction monitoring (MRM) LC–MS method were developed simultaneously, and used to determine saponins and TSs in rat plasma, respectively.

The apparatus used was a Shimadazu LC 20A system consisting of a binary pump, a degasser, an auto-sampler, and a thermostat. Separation was carried out by elution on a Ultimate XB-C18 column (50 mm × 4.6 mm, 3.5 μm). The mobile phase consisted of deionized water–acetic acid (A; 100:0.5, v/v) and acetonitrile (B). The gradient elution was employed as follows: 30–50% B at 0–2 min; 50–80% B at 2–3 min; 80–90% B at 3–4 min; 90% B at 4–9 min; 95% B at 9.1–11 min. The re-equilibrium took 9 min, giving a total run time of 20 min. The flow rate was 0.6 mL/min. The column temperature kept at 30 °C and the volume of sample injected was 2 μL.

Mass spectrometric detection was carried out with a triple quadrupole mass spectrometer (LCMS 8040; Shimadazu Corporation, Japan), operating in the negative electrospray ionization (ESI) mode for saponins and positive ESI mode for TSs. ESI and collision energy were optimized to maximize generation of the characteristic adduct ions, or product ions, respectively. The SIM ions for R_1_, Rg_1_, Rb_1_, Rd, and digoxin were *m/z* 931.35, 859.4, 1107.4, 1005.4, and 779.6, respectively, in negative ionization mode. The precursor-to-product ion pairs used for MRM of DHTS I, CTS, TS I, and TS IIA were *m/z* 279 → 261, 297 → 251, 277 → 249, and 295 → 277 in positive ionization mode, respectively, and digoxin was at *m/z* 839 → 649 in negative ionization mode.

The HPLC–MS method was validated by linearity, limit of quantification (LOQ), limit of detection (LOD), precision, accuracy, extraction recovery, matrix effect, and stability for the eight ingredients, R_1_, Rg_1_, Rb_1_, Rd, DHTS I, CTS, TS I, and TS IIA in plasma. Calibration curves were plotted for the eight ingredients using weighted linear regression of the ratio of analyte and internal standard (digoxin, 500 ng/mL) peak areas against the corresponding nominal concentration of the analyte. LOQ was defined as the lowest concentration at which both precision and accuracy were below 20% with the ratio of signal and noise more than 10 (*S*/*N* > 10). LOD was defined as the detectable concentration at which the ratio of signal and noise was more than 3 (*S*/*N* > 3). Intra- and inter-day accuracy and precision were assessed by detecting of QC samples using six replicates of rat samples at three concentration levels for all the eight ingredients SAA (2, 400, and 1600 ng/mL) on one or three validation days. Accuracy and precision were expressed by relative error (RE) and coefficient of variance (RSD), respectively. The extraction recovery, matrix effect, and stability were determined by the QC samples above-mentioned according to previous reports (Shi et al., 2015; Yang et al., 2016).

### Systems Pharmacology Studies

Firstly, the 3D structures of the ingredients identified with favorable PK properties were drawn, operated by energy minimization (MM2 force fields) and saved as “mol2” type files with Chem 3D Ultra 10.0 software (CambridgeSoftware). All the mol2 type files were submitted to PharmMapper Server (a freely accessed web-server for target fishing, available online at http://lilab.ecust.edu.cn/pharmmapper/index.php) (accessed on March 10, 2018) for searching potential target candidates from 2241 human protein targets for the given small molecules (Liu et al., 2010; Wang et al., 2016; Wang et al., 2017). As a result, predicted top 100 targets ranked by normalized fit score in descending order were obtained from the PharmMapper. Among the targets, those with positive *z*′-score and indicated CVDs relevance were identified as the potential target proteins of the corresponding ingredients, and remained for the following operations. Next, searching the Uniprot databases^3^ to obtain the UniProtKB identifier of each potential target protein and sending the UniProtKB identifiers to MAS 3 (molecule annotation system^4^) to query for the possible CVDs. The resulting proteins were used to construct the C–T–D network graphs using Cytoscape software.^5^ Meanwhile, the target fishing fit scores and correlation degree of target protein in the pharmacology networks corresponding to each ingredient were used to evaluate their possible effect contribution against myocardial ischemia (MI) diseases.

### *In vivo* Anti-MI Study in Mice

The tests were carried out according to our previous report (Yao et al., 2017b) with slight modification. Briefly, mice were randomly allocated into eleven groups (*n* = 8). The first two groups received physiologic saline (0.2 mL, p.o.) and a self-prepared solution (40% PEG-400: 5% Tween-80: water = 54:1:45, 0.2 mL, p.o.) for 7 days and served as sham and model groups, respectively. The remaining nine groups received GDDP, R_1_, Rg_1_, Rb_1_, Rd, DHTS I, CTS, TS I, and TS IIA, respectively (administration solutions were prepared with the self-prepared solution; 0.8 g/kg p.o. dose for GDDP, and 40 mg/kg p.o. dose for others) for 7 days. All groups, except for the sham one, received 5 mg/kg isoprenaline chloride once daily for two successive days (in the fourth and fifth days of treatment). Thirty minutes after administration on the fifth and seventh days of the test, animals were anesthetized with pentobarbital sodium (70 mg/kg; i.p.) for ECG monitoring with standard artifact free lead II (right forelimb to left hind limb). Needle electrodes were inserted subcutaneously into limbs of each mouse and connected to MD3000 bioinformation collector (Huaibei Zhenghua Bioinstrument Co., Ltd., Anhui, China). Twenty-four hours after the last administration, mice were then sacrificed by decapitation. Immediately after the sacrifice of the mice, the hearts treated for sectioning and hematoxylin and eosin (H&E) staining. Sections from the left ventricle were examined by light microscopy (Leica DMR, Germany) at 200× magnification.

### Data Processing

Non-compartmental PK parameters were calculated by DAS 2.0 software (Chinese Pharmacologic Society, Beijing, China). For plasma PK, the area under the plasma concentration–time curve from time zero to the last measurable concentration (AUC_0→t_) was calculated using trapezoidal rule, the AUC_0→∞_ was obtained by extrapolating (AUC_0→t_) to infinity, the apparent elimination half-life (*t*_1/2__β_) was calculated from the terminal log-linear portion of plasma, and the total body clearance (Cl_tot_), apparent volume of distribution (V_d_), mean residence time (MRT), etc. were also calculated by non-compartmental PK mode.

For further comparison by the plasma drug concentration sum method, the plasma drug concentrations of the three markers at each PK time point were summed directly to obtain the proximately total markers concentration in plasma. The resulted total concentration-time curve was prepared by plotting the PK time point against the relative sum concentration. In addition, AUC weighting integrated analysis for the three markers was performed according to the previous reports (Lu et al., 2008; Hao et al., 2009; Yao et al., 2017b).

All the results are expressed as mean ± S.D. A single-tailed Student’s *t*-test was performed in the work.

## Results

### Strategy and Principles for Identifying PK Markers

Before exploring the PK of a TCM, its constituents should be identified and determined, so that the ingredient contents could be sorted and the structure classification of the ingredients could be further discussed. During experiments, the identified PK markers of a TCM should exhibit favorable drug-like properties (Lu et al., 2008; Shi et al., 2018), such as appropriate PK properties (due to suitable solubility in water and intestinal permeability) and desired bioactivities. The selected PK markers should possess appropriate PK properties to suggest that the bioactive PK markers could pass through biological barrier to distribute toward action sites with therapeutic concentration levels after administration of the TCM. Possessing the desired bioactivities is crucial so that the identified PK markers can at least partly reflect the pharmacological effect of the TCM. Meanwhile, systems pharmacology could conveniently dissect the therapeutic effects and mechanisms of multiple components of complex systems (Danhof, 2016), such as herbal medicines or TCMs (Zhang et al., 2015; Zheng et al., 2016). Especially, the connection degree of a certain disease (e.g., MI referring to myocardial infarction, coronary ischemic syndrome, coronary heart disease, angina, ischemia, heart failure, oxidative injury, etc.) in a constructed compounds–targets–diseases (C–T–D) network could be considered as an loose reflection of effect contribution of an ingredient to a certain disease. The fit scores of proteins in target fishing by PharmMapper and the correlation degree of target proteins in C–T–D network could also be considered as a loose index to represent the target proteins’ importance against the certain disease. Therefore, systems pharmacology seems to be able to predict the effect substances base of a TCM against a certain disease, which could also provide important information for understanding or evaluating the therapeutic effect of multiple components of the TCM.

**FIGURE 2:**
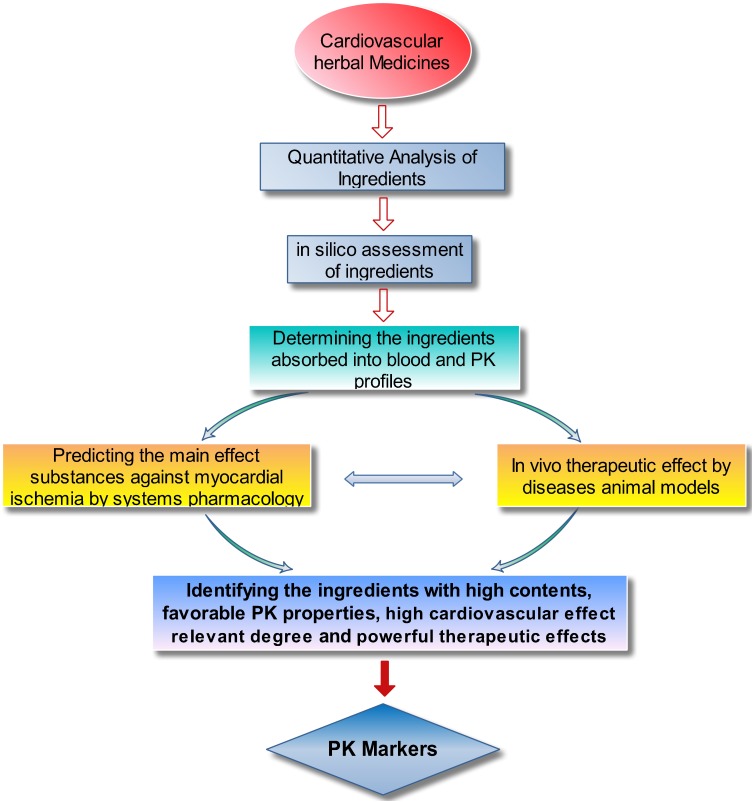
The proposed strategy for identifying PK markers for cardiovascular TCMs.

Considering all the relevant factors, a strategy for identifying PK markers was proposed. As shown in Figure 2, the six main steps are as follows: (1) determining the component constituents and quantifying the chemical ingredients of the studied TCM; (2) sorting and categorizing the ingredients by their contents size and parent structures (e.g., phenolic acids, saponins, and TSs) to determine the ingredients with high content and representative structure feature; (3) *in silico* assessment of the physiochemical properties of the ingredients, including octanol–water partition coefficient (logP), solubility in water (S), topological molecular polar surface area (TPSA), number of hydrogen bond acceptors (nON), and number of hydrogen bond donors (nOHNH), to predict the intestinal permeability and provide reference information for subsequent study; (4) PK profiling of the ingredients absorbed into blood after administration of the TCM to rats by high sensitive and selective analysis methods, such as high-performance liquid chromatography-triple quadrupole mass spectrometry (HPLC–MS/MS); (5) predicting the effect contribution of the ingredients absorbed into blood against MI by systems pharmacology methods and further ascertaining the cardiovascular effects on disease animal models to identify the main effective ingredients in the TCM; and (6) considering the results from the *in silico* assessment, PKs, systems pharmacology, and pharmacological tests, the ingredients with high content, representative structure feature, favorable PK properties (suitable systematic exposure and drawable PK curves), high cardiovascular effect relevant degree, and validated therapeutic effects could be considered the PK markers for the preparation.

### Chemical Components in GDDP

In our previous research (Yao et al., 2017a), an HPLC method was developed to determine the chemicals and their contents in TCM compound preparations containing Danshen–Sanqi herb pair. A total of 17 ingredients were determined in GDDP (Figure 1). According to their structure characterization, these ingredients could be classified into three categorization, *viz*. phenolic acids (including DSS, PCA, PCAL, CA, RA, LA, SAB, and SAA), saponins (including protopanaxatriols R_1_, Rg_1_, and Re and protopanaxatriols Rb_1_ and Rd), and TSs (DHTS I, CTS, TS I, and TS IIA). Among them, Rd (15,146.80 ± 1.92 μg/g), Rg_1_ (6119.50 ± 3.06 μg/g), Rb_1_ (4040.30 ± 1.84 μg/g), TS IIA (3212.17 ± 2.31 μg/g), R_1_ (1221.00 ± 0.38 μg/g), and SAB (1045.00 ± 0.32 μg/g) were higher than others (beyond 0.1% in the preparation). The contents of the remaining ingredients were lower than 0.07%; especially, the PCA (26.40 ± 0.18 μg/g), PCAL (12.30 ± 0.03 μg/g), CA (50.9 ± 0.04 μg/g), and DHTS I (56.6 ± 0.02 μg/g) contents were considerably low (below 0.01%). The content ordering was as follows: Rd > Rg_1_ > Rb_1_ > TS IIA > R_1_ > SAB > CTS > TS I > RA > LA > DSS > Re > SAA > DHTS I > CA > PCA > PCAL (P < 0.05, each ingredient vs. the others). The phenolic acids, saponins, and TSs were approximately 2.41, 26.77, and 4.42 mg/g, respectively. In addition, GDDP also included a major pharmaceutic adjuvant *PEG 6000* (occupying about 70% mass in the preparation) and volatile oil from Jiangxiang. According to the results and considering the low oral administration dosage for human beings (e.g., 0.4 g per administration), the ingredients absorbed into blood could be mainly saponins.

### Molecular Physiochemical Properties

As listed in Table 1, the determined solubility (S) of the eight phenolic acids and five saponins were more than 10 mg/mL in water at 25 °C at 1 atm. However, DHTS I, CTS, TS I, and TS IIA, were insoluble in water at 25 °C, and their calculated S values were 7.7, 9.8, 11.5, and 10.4 μg/mL, respectively. These values suggested their low plasma drug concentrations and poor bioavailability. The calculated LogP (cLogP) values for the four TS ingredients were more than 3, which indicated their high hydrophobicity. The cLogP values for PCA, PCAL, CA, RA, SAA, SAB, Rb_1_ R_1_, Rg_1_, Re, and Rd were between 0 and 3, thereby suggesting their moderate hydrophobicity and hydrophilicity. Moreover, only DSS, PCA, PCAL, CA, DHTS I, CTS, TS I, and TS IIA were favorable on all the values of cLogP, TPSA, MW, nON, and nOHNH according to the Lipinski’s rule of 5 (favorable LogP < 5, TPSA < 140, MW < 500, nON ≤ 10, and nOHNH ≤ 5) (Lipinski et al., 2001). These results possibly indicated their favorable intestinal permeability and drug-likeness. In fact, the *in vitro* permeability for all the 17 ingredients have ever been reported using Caco-2 permeability assay (Lu et al., 2008; Liu et al., 2009; Hu et al., 2014; Bowles et al., 2017). According to the previous results (Table 1), also only DSS, PCA, PCAL, CA, DHTS I, CTS, TS I, and TS IIA showed the apparent permeability of ≥10^-6^ cm/s in Caco-2 cell monolayers and were considered to be favorable for their absorption *in vivo*. However, considering the low solubility or considerably low contents, the plasma concentrations of phenolic acids and TSs could also be low after oral administration of GDDP in rats. Meanwhile, R_1_, Rg_1_, Rb_1_, and Rd, possessed moderate apparent permeability (>10^-7^ cm/s) (Liu et al., 2009), high solubility and contents, suggesting that they could be detected relatively easy in blood after oral administration of the GDDP. These results provided useful reference information for subsequent procedures, such as identifying the absorbable ingredients in plasma and understanding their low systematic exposure levels.

### Multiple-Component PK Profiles for GDDP

Taking the 17 ingredients quantified in GDDP as the observed objects, we identified those absorbed into blood by HPLC–MS/MS with SIM or MRM modes after oral administration of GDDP to rats with a dosage of 0.8 g/kg B.W. (equal to 6.7 times dosage per day to human beings). Thirteen ingredients, including DSS, PCAL, PCA, CA, R_1_, Rg_1_, Re, Rb_1_, Rd, DHTS I, CTS, TS I, and TS IIA, could be detected (LOD, 0.5–0.7 ng/mL) at 5, 10, 15, 30, 45, or 60 min time points in rat plasma after administration. However, among them, only R_1_, Rg_1_, Rb_1_, Rd, DHTS I, CTS, TS I, and TS IIA could be determined (LOQ, 1.0–1.4 ng/mL) at the planned successive PK time points (5, 10, 15, 30, 45, 60, and 120 min). Furthermore, the plasma levels of saponins were more than10-fold higher than those of the TSs. These results agreed with the predictions from the *in silico* assessment and the ingredients’ content size.

For the PK analysis of R_1_, Rg_1_, Rb_1_, Rd, DHTS I, CTS, TS I, and TS IIA in rats, the methodological validation referring to specificity, linearity, LOD, LOQ, precision, accuracy, extraction recovery, matrix effects, and stability, was performed. The typical SIM chromatograms for R_1_, Rg_1_, Rb_1_ and Rd and MRM chromatograms for DHTS I, CTS, TS I, and TS IIA are shown in Supplementary Figures S1 and S2, respectively. No endogenous interference could be observed, which suggested the good specificity of the presented HPLC–MS bioanalysis. Supplementary Table S1 provides a list of the calibration parameters, namely, regression equation, linearity range, *R*^2^, LOD, and LOQ, for the eight ingredients. Results showed good linearity (*R*^2^ > 0.99), wide range (about 1–2600 ng/mL), and excellent LOQs (<1.4 ng/mL) and LODs (<0.7 ng/mL). Supplementary Table S2 lists the results of precision (RSDs < 20%) and accuracy (REs, -18.22 to –12.99%). Results of extraction recoveries and matrix effects are presented in Supplementary Table S3. The sample stabilities after 24 h in autosampler vials, three freeze–thaw cycles, and placing plasma sample for 6 h at 4°C are shown in Supplementary Table S4. These results demonstrated that the method was available for simultaneous determination of the eight ingredients in rat plasma.

**FIGURE 3:**
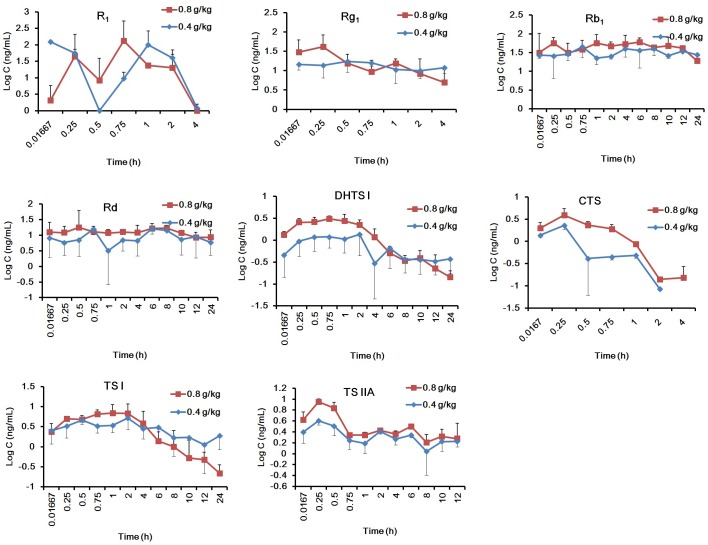
The mean plasma drug concentration–time profiles of the eight ingredients after oral administration of GDDP to rats with the dosages 0.4, and 0.8 g/kg B.W.

The validated method was successfully applied to the PK study of R_1_, Rg_1_, Rb_1_, Rd, DHTS I, CTS, TS I, and TS IIA in rat plasma after intragastric administration of GDDP. The mean plasma drug concentration–time profiles of the ingredients after oral administration of GDDP (0.4 and 0.8 g/kg) to rats are shown in Figure 3.

The main PK parameters were obtained from the non-compartmental mode calculation. As listed in Supplementary Table S5, the systematic exposure to Rb_1_ was the largest, that is, 3.5–30 times higher than those of Rd, R_1_, Rg_1_, TS IIA, and TS I (*P* < 0.05), and 84–1137 times higher than those of CTS and DHTS (*P* < 0.05), according to the AUC_0-∞_. The order of the ingredients based on the AUC_0-∞_ was Rb_1_ > Rd > R_1_ > TS IIA ≥ Rg_1_ ≥ TS I > DHTS I > CTS. According to the *t*_1/2__β_, the ingredients could be classified into two categorizations, namely, fast elimination type (*t*_1/2__β_ < 2 h) including R_1_, Rg_1_, and CTS, and slow elimination type (*t*_1/2__β_ > 2.6 h), including Rb_1_, Rd, TS I, DHTS I, and TS IIA. The order of MRT_0-_*_t_* and MRT_0-∞_ values further supported the classification according to the *t*_1/2__β_ values. In general, the PK behavior of Rg_1_ was similar to those of other protopanaxatriols and that of Rb_1_ was similar to those of other protopanaxadiols. In addition, according to the *C*_max_ values, the ingredients could be classified into two categorizations: with relatively high plasma drug concentrations, including R_1_, Rb_1_, Rd, and Rg_1_ (*C*_max_ > 20 ng/mL), and with low plasma drug concentrations, including TS I, DHTS I, TS IIA, and CTS (*C*_max_ < 10 ng/mL). According to these results from the PK studies, the PK properties of R_1_, Rb_1_, Rd, and Rg_1_ could be more favorable (e.g., easily determined plasma concentration levels and larger AUCs) than those of the TSs.

### Systems Pharmacology Analysis

**FIGURE 4:**
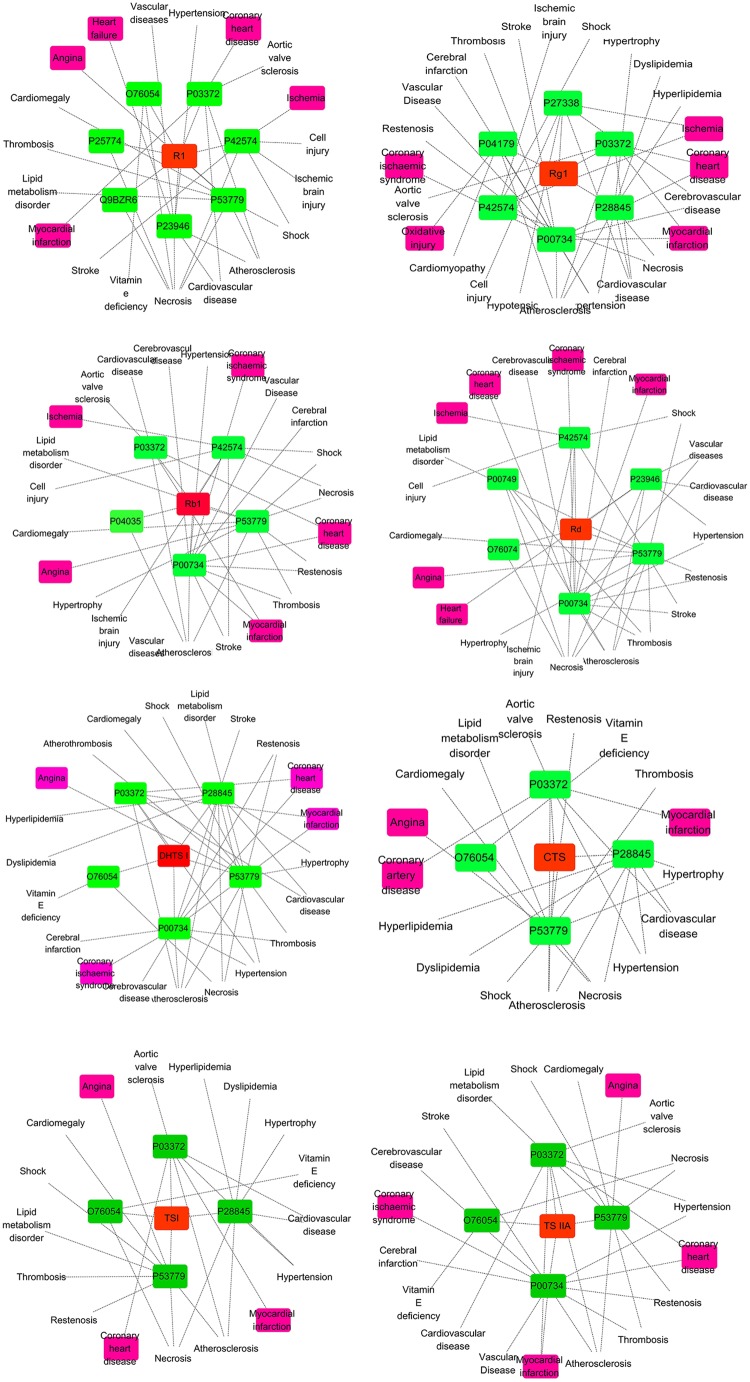
The C–T–D network graphs of systems pharmacology for R_1_, Rb_1_, Rd, Rg_1_, TS I, DHTS I, TS IIA, and CTS against cardiovascular affairs. The purple, green, and red rectangles represent the cardiovascular issues, target proteins, and ingredients, respectively.

During the systems pharmacology investigation, querying MAS 3 (molecule annotation system, see text footnote 4) obtained some potential target proteins which were explicitly marked to be correlated to cardiovascular issues. Among of them, prothrombin (F2, Uniprot ID: P00734), mitogen-activated protein kinase 10 (MAPK10, Uniprot ID: P53779), estrogen receptor (ESR1, Uniprot ID: P03372), caspase-3 (CASP3, Uniprot ID: P42574), chymase (CMA1, Uniprot ID: P23946), amine oxidase B (MAOB, Uniprot ID: P27338), and superoxide dismutase 2 (SOD2, Uniprot ID: P04179) were closely relative to MI issues inferring to myocardial infarction, coronary ischemic syndrome, coronary heart disease, angina, ischemia, heart failure, or oxidative injury. As shown in Figure 4, the eight ingredients, Rg_1_, Rb_1_, Rd, R_1_, TS IIA, DHTS I, CTS, and TS I with favorable PK properties show different therapeutic relevance to the MI issues. The connection frequency (relevance degree) on the MI issues for the eight ingredients above are 8, 7, 6, 5, 6, 4, 3, and 3, respectively, in the C–T–D networks. The loose relationship between the ingredients and the MI diseases inspired us to predict that the therapeutic effects for Rg_1_ and Rb_1_, Rd, TS IIA on MI diseases could be more powerful than those for R_1_, DHTS I, CTS, and TS I.

For further analysis, the fit scores of proteins to the above eight ingredients for target fishing by PharmMapper and the correlation degree of target proteins in the C–T–D networks also listed in Table 2. According to the results, three potential target proteins (closely relative to MI issues), F2, MAPK10, and ESR1 showed high correlation degree in the C–T–D networks. Accordingly, their total values of correlation degree in the C–T–D networks are relatively large (60, 62, and 46, respectively). The sum of fit scores in target fishing by PharmMapper for the studied eight ingredients as to F2, MAPK10, or ESR1 are also relatively high (14.587, 19.86, or 20.226, respectively). The results suggested that the three proteins could be the important targets for the multiple ingredients of GDDP against MI issues. Meanwhile, as to the individual ingredient, the sums of correlation degree/fit scores of the potential targets closely relative to MI issues in the C–T–D networks were 36/16.529, 35/11.855, 37/11.754, 29/11.771, 29/8.174, 26/8.308, 14/5.618, and 17/5.618 for Rg_1_, Rb_1_, Rd, R_1_, TS IIA, DHTS I, CTS, and TS I, respectively. These results further encouraged us to predict that Rg_1_, Rb_1_, and Rd could be the most important therapeutic ingredients to MI diseases, and TS IIA was more powerful than the others three *TS*s, DHTS I, CTS, and TS I in the GDDP.

**Table 2:** The results of systems pharmacology investigation for the eight ingredients in GDPP.

			Correlation degree of target proteins in C–T–D network/fit scores of proteins in target fishing by PharmMapper								
Protein or gene	Uniprot ID	Closely relative diseases to myocardial ischemia	Rg_1_	Rb_1_	Rd	R_1_	CTS	DHI	TS I	TS IIA	Total (all ingredients against one target)
F2	P00734	Myocardial infarction, coronary ischemic syndrome, coronary heart disease	12/2.899	12/2.915	12/3.169	_–_	_–_	12/2.746	_–_	12/2.858	60/14.587
MAPK10	P53779	Angina	_–_	10/2.984	10/2.964	8/2.963	8/2.752	8/2.71	9/2.752	9/2.735	62/19.86
ESR1	P03372	Myocardial infarction, coronary heart disease	6/2.904	6/3.008	_–_	6/3.149	6/2.866	6/2.852	8/2.866	8/2.581	46/20.226
HSD11B1	P28845	^∗^	7/3.155	_–_	_–_	_–_	7/3.142	7/3.805	8/3.142	_–_	29/13.244
CASP3	P42574	Ischemia	7/2.92	7/2.948	7/2.928	7/2.978	_–_	_–_	_–_	_–_	28/11.774
CMA1	P23946	Heart failure	_–_	_–_	8/2.693	8/2.681	_–_	_–_	_–_	_–_	16/5.374
SEC14L2	O76054	^∗^	_–_	_–_	_–_	3/3.972	3/3.61	3/3.636	3/3.61	3/3.502	15/18.33
MAOB	P27338	Ischemia	6/4.359	_–_	_–_	_–_	_–_	_–_	_–_	_–_	6/4.359
SOD2	P04179	Oxidative injury	5/3.447	_–_	_–_	_–_	_–_	_–_	_–_	_–_	5/3.447
PLAU	P00749	^∗^	_–_	_–_	3/3.52	_–_	_–_	_–_	_–_	_–_	3/3.52
PDE5A	O76074	^∗^	_–_	_–_	2/2.954	_–_	_–_	_–_	_–_	_–_	2/2.954
CTSS	P25774	^∗^	_–_	_–_	_–_	2/2.845	_–_	_–_	_–_	_–_	2/2.845
RTN4R	Q9BZR6	^∗^	_–_	_–_	_–_	2/2.511	_–_	_–_	_–_	_–_	2/2.511
HMGCR	P04035	^∗^	_–_	2/3.744	_–_	_–_	_–_	_–_	_–_	_–_	2/3.744
Total (closely relative to myocardial ischemia issues)			36/16.529	35/11.855	37/11.754	29/11.771	14/5.618	26/8.308	17/5.618	29/8.174	


*^∗^Means the others related-cardiovascular affairs excluded.*

*_–_Represents the protein failed to be selected in the target fishing due to negative z′-score by PharmMapper.*

### Protective Effects of the Ingredients on Isoprenaline-Induced MI in Mice

**FIGURE 5:**
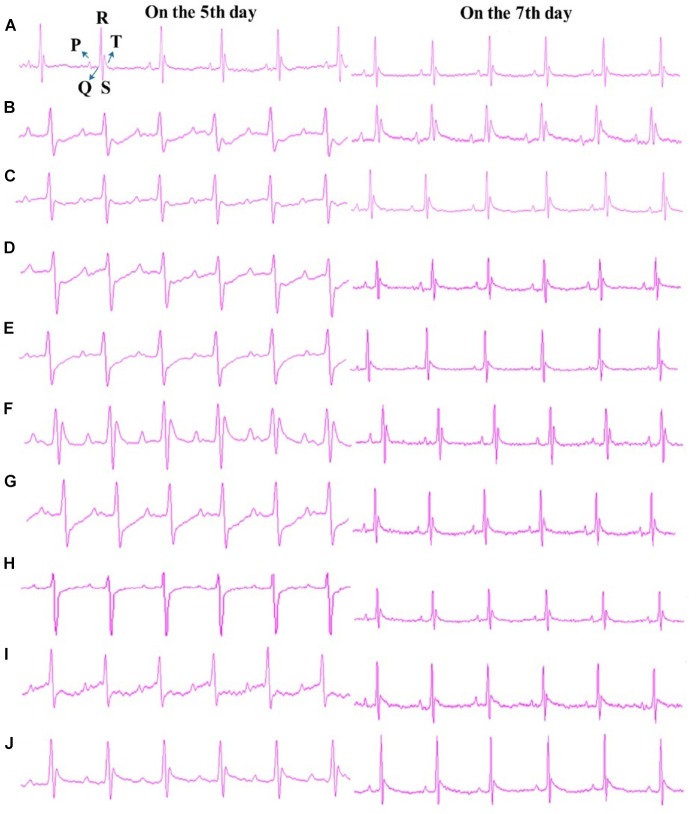
Typical Lead II ECG graphs. **(A)** Sham, **(B)** Model, **(C)** R_1_-treated, **(D)** Rg_1_-treated, **(E)** Rb_1_-treated, **(F)** Rd-treated, **(G)** DHTS I-treated, **(H)** CTS-treated, **(I)** TS I-treated, and **(J)** TS IIA-treated. Dose was 40 mg/kg B.W. for all the treated groups. “On the 5th day” and “On the 7th day” mean that the ECGs were detected on the fifth day and seventh day of the trial, respectively.

R_1_, Rb_1_, Rd, Rg_1_, TS I, DHTS I, TS IIA, and CTS were further examined (40 mg/kg dosage for each; p.o. dosing) in the anti-MI study in mice. As shown in Figure 5A, the ECG of the normal mice showed regular pattern with defined P, QRS, and T waves. Isoprenaline-induced MI mouse model showed abnormal QRS wave and reversed T wave in 30 min after the second injection of isoprenaline to mice (on the fifth day of the trial) and significantly elevated ST segments (*P* < 0.05 vs. sham group) on the seventh day of the trial (Figure 5B). These observations indicated the MI mouse models were prepared successfully. The abnormity of QRS waves occurred in all the eight ingredient-treated groups after modeling; additionally, T waves reversed or decreased in the R_1_, Rg_1_, Rb_1_, DHTS I, CTS, and TS I groups (Figures 5C–H), except for Rd- and TS IIA-treated groups on the fifth day of the trial (ST segment elevated) (Figures 5I,J). As shown in Supplementary Figure S3, the ST segment elevations in Rg_1_-and TS IIA-treated groups were significantly decreased compared with that in model group (*P* < 0.05, vs. MI group) on the seventh day of the trial.

**FIGURE 6:**
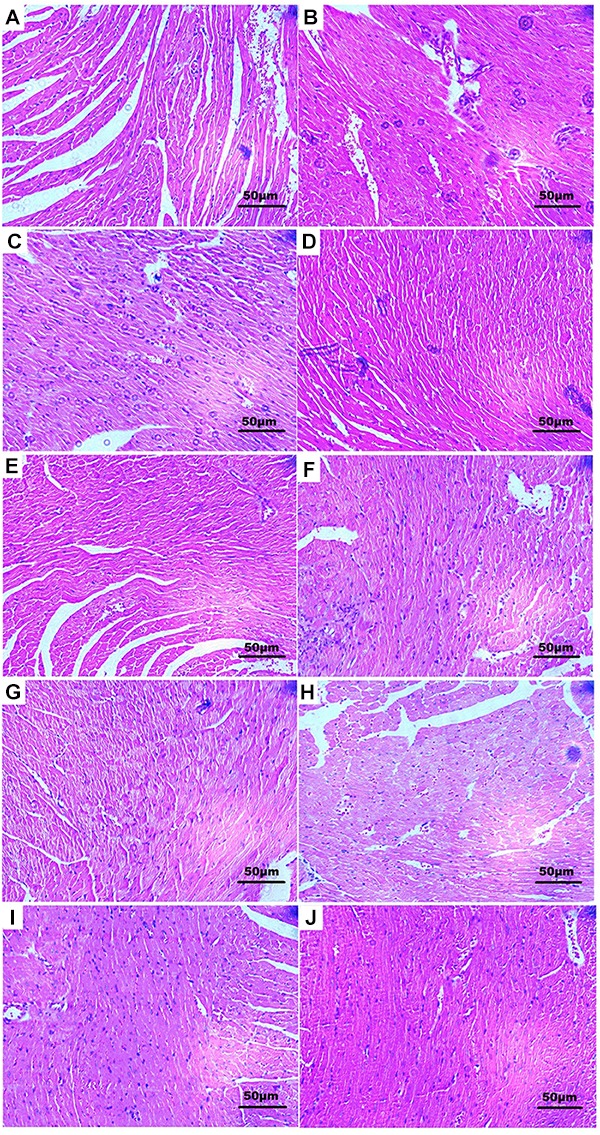
Histological observations of hearts from each group. **(A)** Normal mouse showing normal architecture of the heart tissue with striations, branched appearance, and continuity with adjacent myofibrils; **(B)** isoprenaline-induced MI mouse showing apparent cell distortion, edema, hypochromatosis, and inferior continuity with adjacent myofibrils; **(D)** Rg_1_- and **(E)** Rb_1_-treated mice showing showed less severe histological damage with mild cell distortion, edema, and good continuity with adjacent myofibrils; **(I)** TS I-treated mice showing myocardial lysis, and architecture disorder, and loss of striations in heart tissue; **(J)** TS IIA-treated group showed edema, inflammatory infiltration, and loss of striations in heart tissue. **(C)** R_1_-, **(F)** Rd-, **(G)** DHTS I-, and **(H)** CTS-treated mice showing extremely severe histological damage with extensive myocardial lysis and architecture wrecking. Dose was 40 mg/kg B.W. for all groups.

Histopathology of mouse heart from control group showed a normal architecture structure with striations, branched appearance, fusiform shape, and continuity with adjacent myofibrils (Figure 6A). Heart tissue from the MI mouse models showed apparent cell distortion, edema, hypochromatosis, and inferior continuity with adjacent myofibrils (Figure 6B). Tissue sections from the Rg_1_- and Rb_1_-treated groups showed less severe histological damage, *viz*. slight cell distortion, edema, and good continuity with adjacent myofibrils (Figures 6D,E). TS IIA-treated group showed edema, inflammatory infiltration, and loss of striations in heart tissue (Figure 6J). The TS I-treated group showed myocardial lysis, architecture disorder, and loss of striations in heart tissue (Figure 6I). Nevertheless, all the CTS-,DHTS I-, Rd-, and R_1_-treated (Figures 6H,G,F,C, respectively) groups exhibited remarkably severe histological damage with extensive myocardial lysis and architecture wrecking. These results suggested that Rg_1_ and Rb_1_ possessed favorable anti-MI effect, and TS IIA could also exert mild therapeutic effect against CVDs in GDDP.

### Identifying PK Markers and Integrated PK Investigation for GDDP

Chemical component analysis results pointed out that Rd, Rg_1_, Rb_1_, TS IIA, R_1_, and SAB were the main components in GDDP. Assessment of molecular physiochemical properties showed that R_1_, Rg_1_, Rb_1_, and Rd could be detected relatively easy in blood after oral administration of the GDDP. The multiple-component PK studies further confirmed that R_1_, Rg_1_, Rb_1_, and Rd possessed relatively more favorable PK properties than those of other ingredients in GDDP, although the TSs, DHTS I, CTS, TS I, and TS IIA could also be determined at the studied PK time points. Systems pharmacology deduced Rg_1_, Rb_1_, and Rd could be the important therapeutic ingredients to MI diseases and TS IIA could also be the most important one of active TSs absorbable into circulation system, and they should be further weighed and considered. Evidence from the *in vivo* anti-MI experiments confirmed that only Rg_1_ and Rb_1_ were the most effective components, and TS IIA exhibited mild positive effect against MI injury in MI model mice. Finally, comprehensively considering the content sizes, molecular physiochemical properties, PK characterizations, and therapeutic effects, Rg_1_, Rb_1_, and TS IIA were identified as the PK markers for profiling the *in vivo* process of protopanaxatriols, protopanaxadiols, and TSs, respectively, in GDDP. These three ingredients displayed high content, representative structure feature (protopanaxatriols, protopanaxadiols, and TSs), favorable PK properties, and validated therapeutic effects.

**FIGURE 7:**
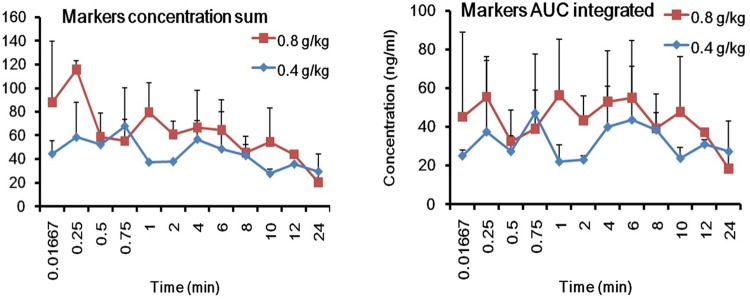
The integrated plasma drug concentration-time curves by the plasma drug concentration sum and AUC weighing integrated methods after single p.o. administration of 0.4 and 0.8 g/kg GDDP (mean ± SD, *n* = 3).

To obtain easy PK parameters, we proposed to carry out the integrated PK studies by using the identified PK markers according to previous reports, *viz*. drug concentration sum method (Yao et al., 2017b) and AUC weighting method (Lu et al., 2008). The total concentrations of the three PK markers in rat plasma were calculated by the two methods at the studied PK time points, respectively. Consequently, the integrated plasma drug concentration–time curves and PK parameters are shown in Figure 7 and Supplementary Table S5, respectively. The outlines of the PK curves by the two integrated methods were approximately similar to each other, and no apparent difference was observed for almost all the PK parameters between the two methods, except for *T*_max_. The AUC weighting integrated method could highlight the contribution of Rb_1_ to the integrated PK parameters and to the integrated PK curve appearance due to its large systematic exposure. However, the plasma drug concentration sum method could be relatively equitable to include the concentration contribution of each PK marker to the integrated PK parameters and curves at each PK time point.

## Discussion

Almost all the previous PK reports on herbal medicines took the constituents being adequate abundance in the herbal products and possessing favorable PK properties as index ones for the PK investigation (Li et al., 2007, 2008; Tong et al., 2010; Duan et al., 2011), but little further considering relative medical effect, because indeed it is unrealistic to examine the pharmacodynamic effect of all unknown and known chemicals coexisting in herbal medicines. Therefore, it would be extremely valuable to put forward and verify a general and feasible strategy for identifying representative PK markers for multicomponent herbal medicines, giving attention to both of favorable PK properties and medical effect for the identified markers. In this study, considered both the PK profiles and the therapeutic effects of the potential PK markers and highlighted the decisive role of the therapeutic effects of potential PK markers with the *in silico* assessment, systems pharmacology, and *in vivo* experimental evidences. Hence, the *in vivo* process of the eight ingredients in GDDP was profiled. This paper is the first to report the PK profiles of the multiple ingredients of GDDP, the obtained PK parameters will be helpful to understand the relationship between administration and the therapeutic effect of GDDP, and the presented strategy will provide a reliable reference for identifying PK markers for other cardiovascular TCMs with the Danshen and Sanqi herbal pair.

As discussed in a recent review about PK study of TCMs (Shi et al., 2018), exploring or representing the whole *in vivo* process of a TCM is a considerable challenge owing to the extremely complex components in a TCM (Alolga et al., 2015). Danshen and Sanqi is a known herb pair contained in many cardiovascular TCM preparations, such as Fufang Danshen dripping pills, Fufang Danshen tablets, Qishenyiqi dripping pills, and Guanxin Pills (Wei et al., 2007; Yao et al., 2017a). One compound preparation containing the Danshen–Sanqi herb pair should be selected as a representative to explore the PK study strategy and represent the *in vivo* process of multiple-component TCMs. Successful attempt should be crucial for the PK study of a series of cardiovascular TCMs containing this herb pair. In recent years, a few efforts in PK investigation of TCMs containing the herb pair or each of them have been reported. Liu et al. (2009) studied the PK properties of multiple ingredients of Sanqi and selected Ra_3_, Rb_1_, and Rd as PK markers for indicating rat systemic exposure to Sanqi extract after oral administration; these markers were selected due to their easily detected blood drug concentration and high systemic exposure level (good PK properties). To identify suitable PK markers that can indicate systemic exposure to compound Danshen Pills, Lu et al. (2008) performed *in vitro* and *in silico* assessments of permeability and solubility and examined *in vivo* PK properties of putatively active phenolic acids, including DSS, PCAL, SAA, SAB, RA, SAD, and LA from the preparation. Consequently, DSS was selected as a PK marker for indicating systemic exposure to the preparation due to the relatively good PK properties of plasma DSS, poor gut permeability, and nearly undetectable levels of other ingredients in plasma and urine in dogs. In addition, Zhang et al. (2012) studied the *in vivo* behavior of multiple ingredients in Qishen Yiqi pills, a TCM compound preparation containing the Danshen–Sanqi pair, Huangqi (*Astragalus membranaceus*), and Jiangxiang. Four putatively active components, namely, DSS, Rg_1_, Rb_1_, and astragaloside IV, were detectable in plasma after oral administration of the preparation (1, 3, and 6 g/kg B.W.) and selected as PK makers to profile the PK behavior of the preparation. Nevertheless, further comparison study should be carried out simultaneously to confirm the therapeutic effects of the identified PK markers.

In the present study, Rg_1_ and Rb_1_ were also detected easily, and they possessed good PK properties. However, DSS could not be detected in rat plasma at more than 15 min after administration of GDDP (0.4 and 0.8 g/kg B.W.). This result could be attributed to that the DSS content in GDDP (0.29 mg/g) was considerably lower than that in Qishen Yiqi pills (14.52 mg/g). Hence, the plasma level of the absorbed DDS was below its LOD (0.5 ng/mL). Moreover, regarding the systematic exposure level, two other saponins, namely, R_1_ and Rd, should be considered in our study. To exclude the unsuitable options, two points must be considered: one is that the ingredients should possess suitable PK profiles, and the other is that the potent therapeutic effects must be verified through comparable experimental studies about the potential PK markers. In our study, the eight ingredients showed the drawable PK curves in the present detection levels (suitable PK properties); among them, only Rg_1_, Rb_1_, and TS IIA showed definitely therapeutic effects against MI, which were accordant to the systems pharmacology prediction. Rg_1_, Rb_1_, and TS IIA should be selected as the PK markers for GDDP because the other ingredients showed no favorable therapeutic effects in our comparison study, regardless of whether they exhibited high systematic exposure level. The PK profiles of multiple ingredients were also pivotal because they confirmed the ingredients that were detectable in blood and could draw PK curves. Furthermore, the PK profiles could provide physiologically relevant basis to the possible therapeutic effects of the components of TCMs (Hao et al., 2014); these profiles are beneficial in scaling out the test objects from the 17 ingredients of GDDP to further confirm their therapeutic effects.

In TCM, four elements, that is, king–minister–assistant–guide, are the basic prescription principles for the individual therapy (Li et al., 2015). Danshen and Sanqi are the main components of GDDP, and they are considered as the king and minister. Jiangxiang is considered as the assistant or guide. In our study, no other ingredient from the component herb Jiangxiang was observed in rat plasma after oral administration of GDDP (0.8 g/kg B.W.). This result could be explained by that the strong liposolubility of volatile oils from Jiangxiang causes low intestinal permeability and absorption (Zhang et al., 2012). As a supposition, Jiangxiang volatile oils could facilitate the absorption of other compounds, thereby their role as the assistant or guide in the formulation.

## Conclusion

In summary, a feasible strategy to identify PK markers for cardiovascular herbal medicines using GDDP as a case was proposed. This study considered both the PK profiles and the therapeutic effects of the potential PK markers and highlighted the decisive role of the therapeutic effects of potential PK markers with the *in silico* assessment, systems pharmacology, and *in vivo* experimental evidences. Hence, the *in vivo* process of the eight ingredients in GDDP was profiled. This paper is the first to report the PK profiles of the multiple ingredients of GDDP. In addition, the integrated PK parameters by the blood drug concentration sum method and AUC weighting method were calculated from the PK curves of the three markers for the first time. All the present findings will be considerably helpful to understand the relationship between administration and the therapeutic effect of GDDP. Finally, the present study will provide a reliable reference for identifying PK markers for other cardiovascular TCMs with the Danshen and Sanqi herbal pair.

## Author Contributions

HY conceived, designed, and performed the experiments, analyzed the data, and wrote the paper. XMH, YX, XLH, and YR performed the experiments and analyzed the data. PS conceived, designed, and performed the experiments, and analyzed the data. XL and LH revised the paper.

## Conflict of Interest Statement

The authors declare that the research was conducted in the absence of any commercial or financial relationships that could be construed as a potential conflict of interest.

## Footnotes

1 http://www.vcclab.org (accessed April 18, 2018)

2 http:/www.molinspiration.com (accessed April 18, 2018)

3 http://www.uniprot.org/uniprot/

4 http://bioinfo.capitalbio.com/mas3/

5 http://www.cytoscape.org/
