# Supplementary material for: Identification of Pharmacokinetic Markers for Guanxin Danshen Drop Pills in Rats by Combination of Pharmacokinetics, Systems Pharmacology, and Pharmacodynamic Assays
Source: Front Pharmacol. 2018 Dec 21;9:1493. doi: 10.3389/fphar.2018.01493 (PMC6308302; doi:10.3389/fphar.2018.01493)
Supplement: Supplementary file 1 [file Data_Sheet_1.doc]

Supplementary Material

**Identification of Pharmacokinetic Markers for Guanxin Danshen Drop Pills in Rats by Combination of Pharmacokinetics, Systems Pharmacology and Pharmacodynamic Assays**

**Hong Yao^1†,*^ Xiaomei Huang^1†^,Yunjiao Xie^1^, Xuliang Huang^1^, Yijun Ruan^1^, Xinhua Lin^1^, Liying Huang^1^, Peiying Shi^2*^**

† These authors contributed equally to this work.

*** Correspondence:** Hong Yao: [yauhung@126.com](mailto:yauhung@126.com); Peiying Shi: [peiyshi@126.com](mailto:peiyshi@126.com)

# Supplementary Figures


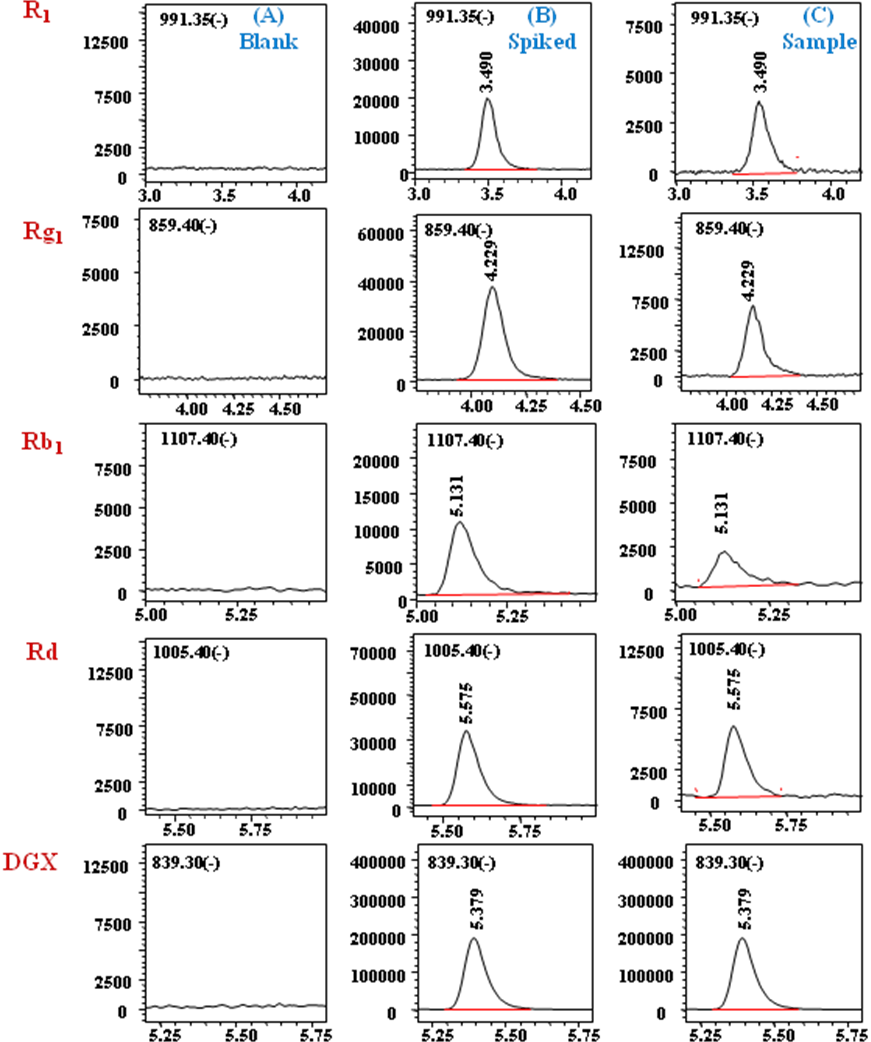


**Supplementary Figure 1.** The typical SIM chromatograms in negative mode for the blank plasma (A), the analytes spiked in the blank plasma (B), the plasma samples of 2 hours after dosing of GDDP (C). R_1_, notoginsenoside R_1_; Rg_1_, ginsenoside Rg_1_; Rb_1_, ginsenoside Rb_1_; Rd, ginsenoside Rd; DGX, Digoxin, internal standard.


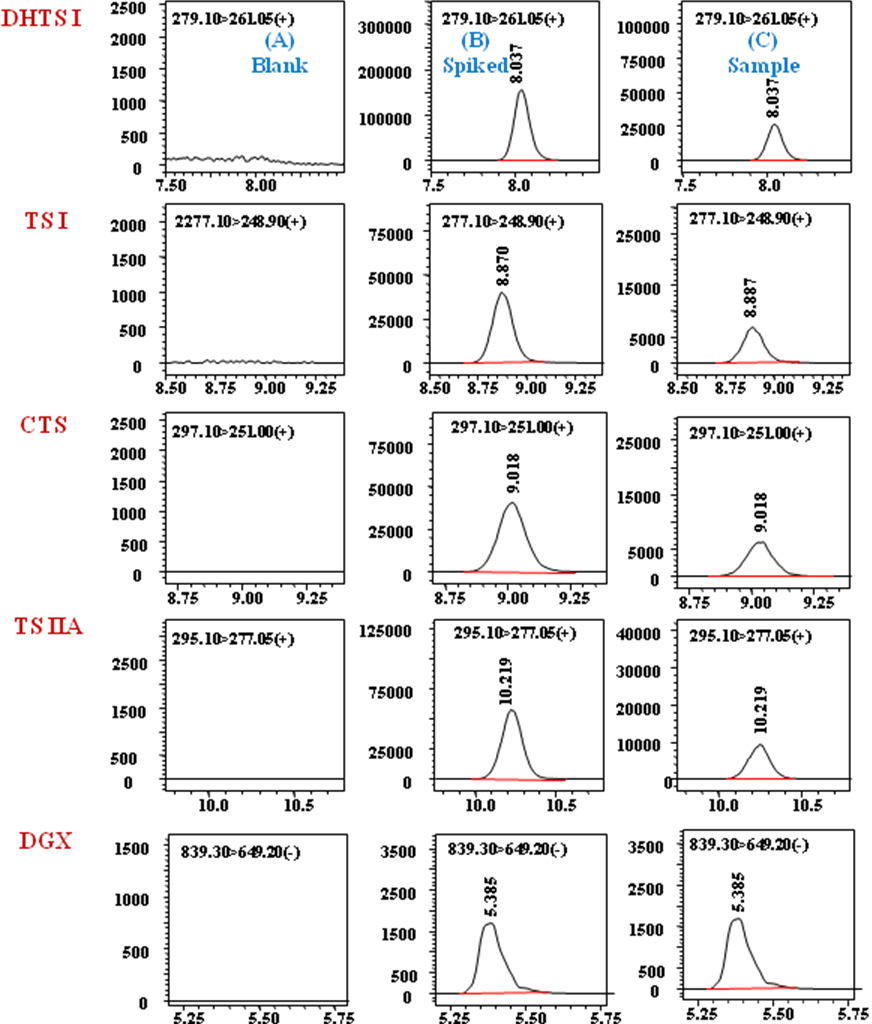


**Supplementary Figure 2.** The typical MRM chromatograms in positive mode for the blank plasma (A), the analytes spiked in the blank plasma (B), the plasma samples of 2 hours after dosing of GDDP (C). DHTS I, dihydrotanshinone I; CTS, cryptotanshinone; TS I, tanshinone I ; TS IIA; DGX, Digoxin, internal standard.


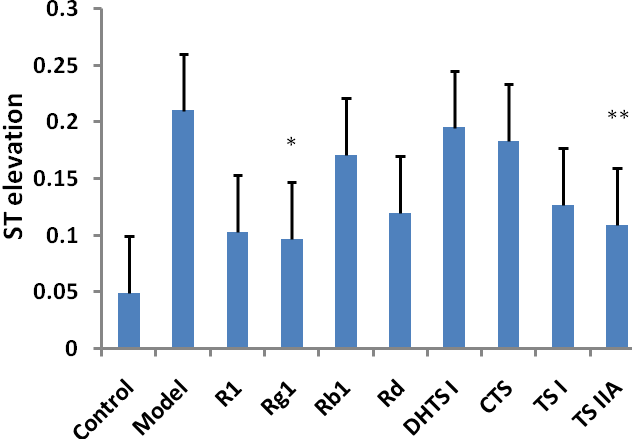


**Supplementary Figure 3.** The ST segament elevations on the 7^th^ of trial. *^*^ P* < 0.05, vs. Control group; *^**^* *P* < 0.05, vs. model group.

# Supplementary Tables

**Supplementary Table** **1.** Calibration parameters for the 8 ingredients in rat plasma.

| Analytes | Calibration curves | Linear range  (ng/mL) | R^2^ | LOD  (ng/mL) | LOQ  (ng/mL) |
| --- | --- | --- | --- | --- | --- |
| R_1_ | y=0.115212x+0.00415262 | 1.32-2640 | 0.99959 | 0.66 | 1.32 |
| Rg_1_ | y=0.210320x+0.00490841 | 1.25-2500 | 0.99911 | 0.625 | 1.25 |
| Rb_1_ | y=0.0485635x+0.00154169 | 1.34-2680 | 0.99610 | 0.67 | 1.34 |
| Rd | y=0.139742x+0.000170069 | 1.12-2240 | 0.99985 | 0.56 | 1.12 |
| DHTS I | y=89.2972x+1.1.19978 | 1.08-2160 | 0.99929 | 0.54 | 1.08 |
| CTS | y=30.0433x+0.00267861 | 1.21-2420 | 0.99847 | 0.605 | 1.21 |
| TS I | y=22.0246x+0.722156 | 1.02-2040 | 0.99755 | 0.51 | 1.02 |
| TS IIA | y=50.1971x+0.129379 | 1.04-2080 | 0.99827 | 0.52 | 1.04 |

**Supplementary Table 2.** Precision, and accuracy for the 8 ingredients in rat plasma.

| Analytes | Spiked (ng/ml) | **Intra-day (n=5)** | | | **Inter-day (n=3)** | | |
| --- | --- | --- | --- | --- | --- | --- | --- |
|  |  | Measured (ng/mL) | Precision  (RSD, %) | Accuracy  (RE, %) | Measured (ng/mL) | Precision  (RSD, %) | Accuracy  (RE, %) |
| R_1_ | 2 | 2.11±0.20 | 9.41 | 5.60 | 1.88±0.24 | 12.73 | -6.00 |
|  | 400 | 353.43±13.70 | 3.88 | -11.64 | 394.13±42.50 | 10.78 | -1.47 |
|  | 1600 | 1444.72±80.45 | 5.57 | -9.70 | 1755.88±220.61 | 12.56 | 9.74 |
| Rg_1_ | 2 | 1.77±0.20 | 11.26 | -11.32 | 1.77±0.12 | 6.68 | -11.33 |
|  | 400 | 387.13±10.10 | 2.61 | -3.22 | 428.18±29.70 | 6.94 | 7.04 |
|  | 1600 | 1558.83±93.96 | 6.03 | -2.60 | 1807.80±181.69 | 10.05 | 12.99 |
| Rb_1_ | 2 | 1.72±0.23 | 13.47 | -14.08 | 1.83±0.11 | 6.01 | -8.50 |
|  | 400 | 367.31±10.71 | 2.92 | -8.17 | 410.77±43.46 | 10.58 | 2.69 |
|  | 1600 | 1489.00±86.37 | 5.80 | -6.94 | 1702.06±213.06 | 12.52 | 6.38 |
| Rd | 2 | 1.64±0.32 | 19.53 | -18.22 | 1.84±0.07 | 3.77 | -7.83 |
|  | 400 | 406.49±13.64 | 3.36 | 1.62 | 413.91±11.42 | 2.76 | 9.23 |
|  | 1600 | 1431.78±73.94 | 5.16 | -10.51 | 1621.49±138.43 | 8.54 | 1.34 |
| DHTS I | 2 | 1.83±0.30 | 16.32 | -8.51 | 1.84±0.13 | 7.11 | -7.83 |
|  | 400 | 408.29±52.33 | 12.82 | 2.07 | 436.91±21.21 | 4.85 | 9.23 |
|  | 1600 | 1424.44±101.29 | 7.11 | -10.97 | 1480.76±56.31 | 3.8 | -7.45 |
| CTS | 2 | 1.76±0.29 | 16.75 | -12.07 | 1.78±0.10 | 5.35 | -10.83 |
|  | 400 | 356.54±12.57 | 3.52 | -10.87 | 416.45±43.73 | 10.5 | 4.11 |
|  | 1600 | 1354.54±125.51 | 9.27 | -15.34 | 1681.74±239.31 | 14.23 | 5.11 |
| TS I | 2 | 2.13±0.26 | 12.31 | 6.40 | 1.91±0.81 | 9.64 | -4.67 |
|  | 400 | 392.64±39.40 | 10.03 | -1.84 | 417.61±23.47 | 5.62 | 4.40 |
|  | 1600 | 1314.31±118.46 | 9.01 | -17.86 | 1341.69±19.44 | 1.45 | -16.14 |
| TS IIA | 2 | 2.06±0.33 | 16.220 | 3.130 | 1.94±0.13 | 6.93 | -3.17 |
|  | 400 | 388.22±45.00 | 11.590 | -2.950 | 401.63±18.90 | 4.71 | 0.41 |
|  | 1600 | 1344.86±119.30 | 8.870 | -15.950 | 1414.34±89.41 | 6.32 | -11.60 |

**Supplementary Table 3.** The results of extraction recoveries and matrix effects (n=3).

| Analytes | Spiked  (ng/mL) | Recovery  (mean ± SD) | Matrix effect  (mean ± SD) |
| --- | --- | --- | --- |
| R_1_ | 2 | 84.10±9.73 | 92.28±20.45 |
|  | 400 | 98.43±2.39 | 99.89±4.00 |
|  | 1600 | 109.39±5.99 | 94.77±3.32 |
| Rg_1_ | 2 | 64.51±1.31 | 92.09±9.88 |
|  | 400 | 97.37±3.44 | 99.73±2.11 |
|  | 1600 | 94.61±5.91 | 98.59±4.40 |
| Rb_1_ | 2 | 101.18±7.04 | 93.67±7.03 |
|  | 400 | 82.89±9.17 | 96.39±0.11 |
|  | 1600 | 116.37±7.67 | 94.27±11.38 |
| Rd | 2 | 75.04±24.19 | 82.31±17.14 |
|  | 400 | 95.46±4.74 | 97.96±2.10 |
|  | 1600 | 96.51±2.11 | 97.28±5.46 |
| DHTS I | 2 | 112.28±35.98 | 91.78±3.52 |
|  | 400 | 79.32±5.72 | 116.82±15.29 |
|  | 1600 | 81.41±14.38 | 114.34±25.52 |
| CTS | 2 | 57.82±23.71 | 98.44±58.24 |
|  | 400 | 83.13±4.91 | 108.78±13.59 |
|  | 1600 | 86.49±15.65 | 106.31±23.02 |
| TS I | 2 | 74.71±28.39 | 106.23±27.48 |
|  | 400 | 77.70±3.83 | 114.96±17.78 |
|  | 1600 | 82.08±14.08 | 112.78±25.02 |
| TS IIA | 2 | 112.04±13.79 | 104.07±16.57 |
|  | 400 | 83.54±3.86 | 111.98±16.26 |
|  | 1600 | 86.07±15.35 | 107.13±22.38 |

**Supplementary Table 4.**  The results for stability tests in rat plasma (n=5).

| Analytes | Spiked (ng/mL) | **24h in auto-sampler vials** | | | **three freeze-thaw cycles** | | | **Placing plasma for 6h** | | |
| --- | --- | --- | --- | --- | --- | --- | --- | --- | --- | --- |
|  |  | Measured (ng/mL) | RSD  (%) | RE  (%) | Measured (ng/mL) | RSD  (%) | RE  (%) | Measured  (ng/mL) | RSD  (%) | RE  (%) |
| R_1_ | 2 | 1.89±0.51 | 26.94 | -5.63 | 2.15±0.21 | 9.65 | 7.54 | 1.75±0.34 | 19.67 | -12.56 |
|  | 400 | 395.76±7.32 | 1.85 | -1.06 | 395.28±15.09 | 3.82 | -1.18 | 387.55±18.40 | 4.75 | -3.11 |
|  | 1600 | 1619.10±82.22 | 5.08 | 1.19 | 1784.04±71.20 | 3.99 | 11.50 | 1698.54±105.94 | 6.24 | 6.16 |
| Rg_1_ | 2 | 1.69±0.24 | 14.49 | -15.63 | 1.75±0.23 | 12.93 | -12.60 | 1.75±0.30 | 17.17 | -12.46 |
|  | 400 | 433.65±26.28 | 6.06 | 8.41 | 456.97±15.97 | 3.49 | 14.24 | 457.68±22.23 | 4.86 | 14.42 |
|  | 1600 | 1628.29±28.27 | 1.74 | 1.77 | 1895.10±83.54 | 4.50 | 15.94 | 1841.18±22.14 | 1.20 | 15.07 |
| Rb_1_ | 2 | 1.97±0.29 | 14.57 | -1.62 | 1.75±0.23 | 13.24 | -12.42 | 1.66±0.17 | 10.48 | -16.86 |
|  | 400 | 386.10±20.53 | 5.32 | -3.47 | 394.28±21.32 | 5.42 | -1.43 | 376.87±18.97 | 5.03 | -5.78 |
|  | 1600 | 1540.64±55.53 | 3.60 | -3.71 | 1601.88±26.56 | 1.66 | 0.12 | 1647.48±31.81 | 1.93 | 2.97 |
| Rd | 2 | 1.93±0.23 | 12.16 | -3.59 | 1.81±0.18 | 9.82 | -9.59 | 1.65±0.33 | 19.75 | -17.56 |
|  | 400 | 406.43±27.13 | 6.68 | 1.61 | 438.01±11.23 | 2.56 | 9.50 | 449.87±18.67 | 4.15 | 12.47 |
|  | 1600 | 1489.53±52.70 | 3.54 | -6.90 | 1768.72±60.05 | 3.40 | 10.54 | 1891.74±134.38 | 7.10 | 18.23 |
| DHTS I | 2 | 2.21±0.23 | 10.30 | 10.31 | 2.015±0.18 | 8.64 | 2.73 | 2.23±0.06 | 2.62 | 11.41 |
|  | 400 | 464.67±43.51 | 9.36 | 16.17 | 437.97±37.90 | 8.65 | 9.49 | 456.85±38.51 | 8.43 | 14.21 |
|  | 1600 | 1644.43±109.72 | 6.67 | 2.78 | 1614.13±306.Z8 | 18.98 | 0.88 | 1735.14±230.11 | 13.26 | 8.45 |
| CTS | 2 | 1.79±0.36 | 20.28 | -10.32 | 1.62±0.22 | 13.60 | -19.00 | 1.79±0.26 | 1.26 | -10.47 |
|  | 400 | 478.43±27.22 | 5.69 | 19.61 | 457.79±26.04 | 5.69 | 14.45 | 438.37±50.69 | 11.56 | 9.59 |
|  | 1600 | 1852.38±164.86 | 8.90 | 15.77 | 1871.43±321.89 | 17.20 | 16.96 | 1890.21±223.22 | 11.81 | 18.14 |
| TS I | 2 | 2.02±0.32 | 15.72 | 0.78 | 2.09±0.20 | 9.64 | 4.39 | 2.22±0.30 | 13.52 | 10.90 |
|  | 400 | 470.56±54.19 | 11.52 | 17.64 | 459.00±25.68 | 5.60 | 14.75 | 469.39±24.45 | 5.21 | 17.35 |
|  | 1600 | 1562.58±145.90 | 9.34 | -2.34 | 1482.38±283.06 | 19.10 | -7.32 | 1771.82±333.46 | 18.82 | 10.74 |
| TS IIA | 2 | 2.25±0.34 | 15.12 | 12.73 | 2.10±0.23 | 11.000 | 4.94 | 2.14±0.11 | 5.18 | 6.84 |
|  | 400 | 464.77±53.87 | 11.59 | 16.19 | 468.67±61.03 | 12.810 | 19.13 | 462.76±13.70 | 2.96 | 15.69 |
|  | 1600 | 1714.76±174.65 | 10.18 | 7.17 | 1830.46±289.25 | 15.800 | 14.40 | 1933.47±224.22 | 11.60 | 20.84 |

**Supplementary Table 5.** The PK parameters of the 8 ingredients, markers concentration sum, or AUC weighting method after oral administration of 0.4g/kg (Low dose group), and 0.8g/kg (High dose group) of GDDP to rats (mean ± SD, n=3).

| Anlaytes | Contents in GDDP(mg/g) | Groups | t_1/2 β_ (h) | T_max_ (h) | C_max_  (ng/mL) | Cltot(L/h/kg) | AUC_0-t_  (ng·h/mL) | AUC_0-∞_  (ng·h/mL) | MRT_0-t_ (h) | MRT_0-∞_ (h) |
| --- | --- | --- | --- | --- | --- | --- | --- | --- | --- | --- |
| R_1_ | 1.221 | Low | 0.57±0.00 | 0.51±0.70 | 166.57±47.98^**^ | 3.34±1.81 | 146.20±58.88 | 171.25±92.60 | 1.10±0.12 | 1.31±0.11 |
|  |  | High | 0.45±0.00 | 0.75±0.00 | 258.73±342.18^**^ | 10.89±11.27 | 119.99±117.06 | 167.74±119.04 | 0.89±0.14 | 1.92±1.59 |
| Rg_1_ | 6.120 | Low | 2.15±2.06 | 0.38±0.18 | 25.47±2.85 ^**^ | 30.60±7.85 | 50.57±2.59 | 82.71±21.21 | 1.88±0.05 | 4.07±2.07 |
|  |  | High | 0.88±0.38 | 0.09±0.13 | 52.90±25.29 ^**^ | 81.02±8.44 | 54.66±8.97 | 60.88±6.56 | 1.35±0.23 | 1.75±0.43 |
| Rb_1_ | 4.040 | Low | 22.95±12.37 ^*^ | 3.25±3.89 | 56.96±27.27 ^**^ | 1.08±0.72 | 817.76±271.52 | 1933.01±1295.80 ^Δ^ | 11.42±0.11 ^*^ | 36.59±17.68 ^*^ |
|  |  | High | 10.82±4.03 ^*^ | 3.34±3.05 | 80.34±19.21 ^**^ | 2.59±0.81 | 997.92±200.31 | 1350.52±485.65 ^Δ^ | 9.63±0.60 ^*^ | 17.08±4.98 ^*^ |
| Rd | 15.15 | Low | 5.63±5.88 | 9.00±4.24 | 24.53±4.04 ^**^ | 37.45±38.33 | 253.75±235.93 | 339.79±347.80 | 8.88±3.79 ^*^ | 12.84±8.78 ^*^ |
|  |  | High | 8.53±4.42 ^*^ | 2.25±3.25 | 37.18±26.27 ^**^ | 21.02±18.63 | 280.75±28.14 | 377.54±56.43 | 10.74±2.31 ^*^ | 17.27±2.01 ^*^ |
| DHTS I | 0.0566 | Low | 2.68±2.38 ^*^ | 1.38±0.88 | 1.90±1.57 | 6.05±7.34 | 12.08±14.40 | 14.16±17.18 | 5.17±5.21 | 6.93±7.38 |
|  |  | High | 5.57±2.98 ^*^ | 0.83±0.14 | 3.10±0.49 | 2.86±0.46 | 14.68±2.51 | 16.15±2.84 | 5.05±2.03 ^*^ | 7.26±3.47 ^*^ |
| CTS | 0.6650 | Low | 0.45±0.45 | 0.25±0.00 | 2.33±0.49 | 209.12±151.07 | 0.84±0.03 | 1.72±1.24 | 0.23±0.03 | 0.69±0.66 |
|  |  | High | 0.57±0.26 | 0.25±0.00 | 4.09±1.47 | 175.44±49.57 | 2.76±1.18 | 3.24±1.09 | 0.56±0.22 | 0.84±0.30 |
| TS I | 0.4899 | Low | 16.38±8.87 ^*^ | 1.25±1.06 | 6.10±3.08 | 2.72±2.51 | 62.97±49.52 | 124.98±115.11 | 9.93±0.87 ^*^ | 27.29±10.59 ^*^ |
|  |  | High | 11.72±4.78 ^*^ | 1.25±0.66 | 8.59±3.18 | 9.78±3.59 | 40.10±17.30 | 45.06±20.55 | 4.27±1.62 ^*^ | 7.72±2.17 ^*^ |
| TS IIA | 3.212 | Low | 18.14±12.52 ^*^ | 0.25±0.00 | 4.04±0.72 | 22.93±15.15 | 23.53±6.40 | 71.69±47.37 | 5.40±0.41 ^*^ | 27.09±16.67 ^*^ |
|  |  | High | 18.31±13.01^*^ | 0.33±0.14 | 9.21±0.78 | 35.11±18.25 | 31.05±1.74 | 90.71±52.89 | 5.23±0.04 ^*^ | 26.52±19.83 ^*^ |
| AUC integrated |  | Low | 29.02±3.75 | 3.24±3.88 | 53.00±28.00 | 3.28±1.86 | 754.13±299.70 | 1941.37±1101.58 | 11.37±0.15 | 43.68±7.54 |
|  |  | High | 16.45±4.78 | 1.42±2.24 | 73.25 ±21.60 | 8.61±2.67 | 900.26±288.89 | 1347.59±506.86 | 9.59±0.59 | 21.92±5.81 |
| Con. sum |  | Low | 25.18±2.03 | 0.63±0.18 | 72.03±28.93 | 2.91±1.05 | 1102.49±188.53 | 1969.93±712.50 | 10.6±0.41 | 36.81±0.67 |
|  |  | High | 12.84±2.64 | 0.17±0.13 | 122.30 ±6.42 | 7.49±1.82 | 914.50±255.99 | 1495.61±419.73 | 9.07±0.53 | 17.56±4.05 |

^*^ *P* < 0.05, vs. R_1_, Rg_1_, or CTS; ^**^ *P* < 0.05, vs. TS I, DHTS I, TS IIA, or CTS; ^Δ^ *P* < 0.05, vs. the other seven ingredients
